# Immunogenicity of a novel tetravalent vaccine formulation with four recombinant lipidated dengue envelope protein domain IIIs in mice

**DOI:** 10.1038/srep30648

**Published:** 2016-07-29

**Authors:** Chen-Yi Chiang, Chien-Hsiung Pan, Mei-Yu Chen, Chun-Hsiang Hsieh, Jy-Ping Tsai, Hsueh-Hung Liu, Shih-Jen Liu, Pele Chong, Chih-Hsiang Leng, Hsin-Wei Chen

**Affiliations:** 1 National Institute of Infectious Diseases and Vaccinology, National Health Research Institutes, 35 Keyan Road, Zhunan, Miaoli 350, Taiwan, Republic of China; 2 Graduate Institute of Immunology, China Medical University, Taichung, Taiwan, Republic of China

## Abstract

We developed a novel platform to express high levels of recombinant lipoproteins with intrinsic adjuvant properties. Based on this technology, our group developed recombinant lipidated dengue envelope protein domain IIIs as vaccine candidates against dengue virus. This work aims to evaluate the immune responses in mice to the tetravalent formulation. We demonstrate that 4 serotypes of recombinant lipidated dengue envelope protein domain III induced both humoral and cellular immunity against all 4 serotypes of dengue virus on the mixture that formed the tetravalent formulation. Importantly, the immune responses induced by the tetravalent formulation in the absence of the exogenous adjuvant were functional in clearing the 4 serotypes of dengue virus *in vivo*. We affirm that the tetravalent formulation of recombinant lipidated dengue envelope protein domain III is a potential vaccine candidate against dengue virus and suggest further detailed studies of this formulation in nonhuman primates.

Dengue disease is a serious public health problem around the world. There are 4 antigenically different serotypes of dengue virus (dengue-1 to dengue-4). Infection with any serotypes of dengue virus can be either asymptomatic or lead to a wide range of diseases from mild dengue fever to life-threatening dengue hemorrhagic fever and dengue shock syndrome^1^. Dengue virus occurs in more than 120 countries through tropical and subtropical areas^2^ and imposes health threats to more than 2 billion people^3^. An up-to-date estimation states that there were 390 million dengue infections globally in 2010^4^. Co-circulation of multiple dengue virus serotypes was observed in the same spatial region^5^,^6^,^7^,^8^,^9^,^10^,^11^ and believed to be an important risk factor for this severe form of disease^1^,^12^. Therefore, an effective vaccine must be a tetravalent formulation against all 4 serotypes of dengue virus.

Several tetravalent dengue vaccines are in clinical development^13^. The most advanced vaccine candidate is led by Sanofi Pasteur, which is based on live attenuated chimeric virus. This vaccine candidate was approved by Mexico at the end of 2015 and became the first dengue vaccine in the world. However, the vaccine efficacies were approximately 60% after three injections in two phase 3 studies^14^,^15^. Available data from long-term follow-up interim analyses reveal that there was an increased risk of hospitalization for dengue in year 3 among children younger than 9 years of age^16^. More studies are required to understand the risk-benefit profile in this age group. Development of a tetravalent dengue vaccine is still a huge challenge.

Our group focuses on dengue envelope protein domain III (ED III) to develop subunit vaccines. ED III is a potential target for dengue vaccine development because ED III is involved in cellular receptor recognition^17^,^18^,^19^ and contains several neutralizing epitopes^18^,^20^,^21^. Several ED III-based subunit vaccines have been evaluated in mice^22^,^23^,^24^,^25^,^26^,^27^,^28^ and nonhuman primates^22^,^29^,^30^,^31^,^32^,^33^. All subunit vaccine candidates cannot induce robust immune responses without exogenous adjuvant formulation. It is unfortunate that alum adjuvant alone, the most widely used in human vaccines, is incompetent to elicit complete protection against dengue virus infection^26^,^29^,^32^. A strategy that is either exogenous adjuvant-free or immunogens with intrinsic adjuvant may provide an alternative for dengue subunit vaccine development.

It has been shown that microbial lipoproteins are potent stimulators of IL-12 production by human macrophages through toll-like receptors. Activation of the toll-like receptors by microbial lipoproteins may trigger innate defense mechanisms against infectious pathogens^34^. In our previous studies, we found a suitable *Escherichia coli* strain to express lipoprotein in high yields. In addition, non-lipoprotein could be converted into lipoprotein by fusion with a fragment of the Ag473 lipoprotein of *Neisseria meningitidis*^35^. We demonstrated that *Escherichia coli*-expressed lipoprotein stimulated innate immunity through the toll-like receptor 2^36^ and induced superior adaptive immunity to its non-lipidated counterpart^35^,^37^. This lipidation approach has been applied to each serotype of ED III^38^,^39^,^40^,^41^, but a tetravalent formulation has not yet been characterized. In the present study, we further analyzed the profiles of T-cell epitopes and antibody responses in mice after immunization with tetravalent lipidated ED III (tLED III). Our results indicate that functional immune responses were generated in tLED III-immunized mice and suggest that tLED III is a potential tetravalent dengue vaccine candidate.

## Results

### The tetravalent formulation induces durable and broad profile of humoral immuneresponses in mice

**Figure 1:**
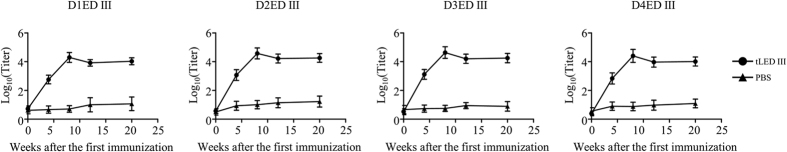
Induction of durable antibody responses in mice immunized with a tetravalent recombinant lipidated dengue envelope protein domain III. Groups of BALB/c mice were immunized subcutaneously twice with a tetravalent vaccine (10 μg of each serotype of recombinant lipidated dengue envelope protein domain III) or PBS at a 4-week interval. Serum samples were collected at 4, 8, 12, and 20 weeks after the first immunization. IgG antibodies against D1ED III, D2ED III, D3ED III, and D4ED III were evaluated by ELISA, respectively. Data represent the mean ± standard deviation from total 12 mice of 2 experiments.

**Figure 2:**
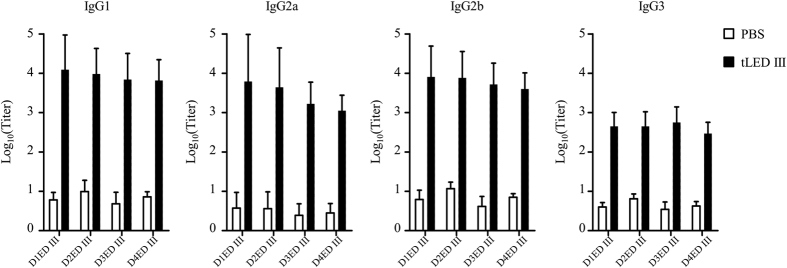
Analysis of IgG antibody subclasses in mice immunized with a tetravalent recombinant lipidated dengue envelope protein domain III. Groups of BALB/c mice were immunized subcutaneously twice with a tetravalent vaccine (10 μg of each serotype of recombinant lipidated dengue envelope protein domain III) or PBS at a 4-week interval. Serum samples were collected 8 weeks after the first immunization. IgG1, IgG2a, IgG2b, and IgG3 antibodies against D1ED III, D2ED III, D3ED III, and D4ED III were evaluated by ELISA, respectively. Data represent the mean ± standard deviation from total 12 mice of 2 experiments.

Groups of BALB/c mice were immunized with PBS or tLED III two times at a 4-week interval and monitored for antibody responses. Serum samples were collected at weeks 4, 8, 12, 20 after the primary immunization and tested for antibody responses against ED III of all 4 serotypes. As shown in Fig. 1, tLED III induced equally high ED III-specific titers against all 4 serotypes. Antibody titers were increased after a booster immunization and were held for at least 20 weeks after the primary immunization. Serum samples which were collected 8 weeks after the primary immunization was further analyzed in the ED III-specific IgG subtypes. All the ED III-specific IgG subtypes (IgG1, IgG2a, IgG2b, and IgG3) were generated in mice immunized with tLED III (Fig. 2). Antibody titers of each IgG subtype were equivalent among the four dengue envelope protein domain IIIs.

**Figure 3:**
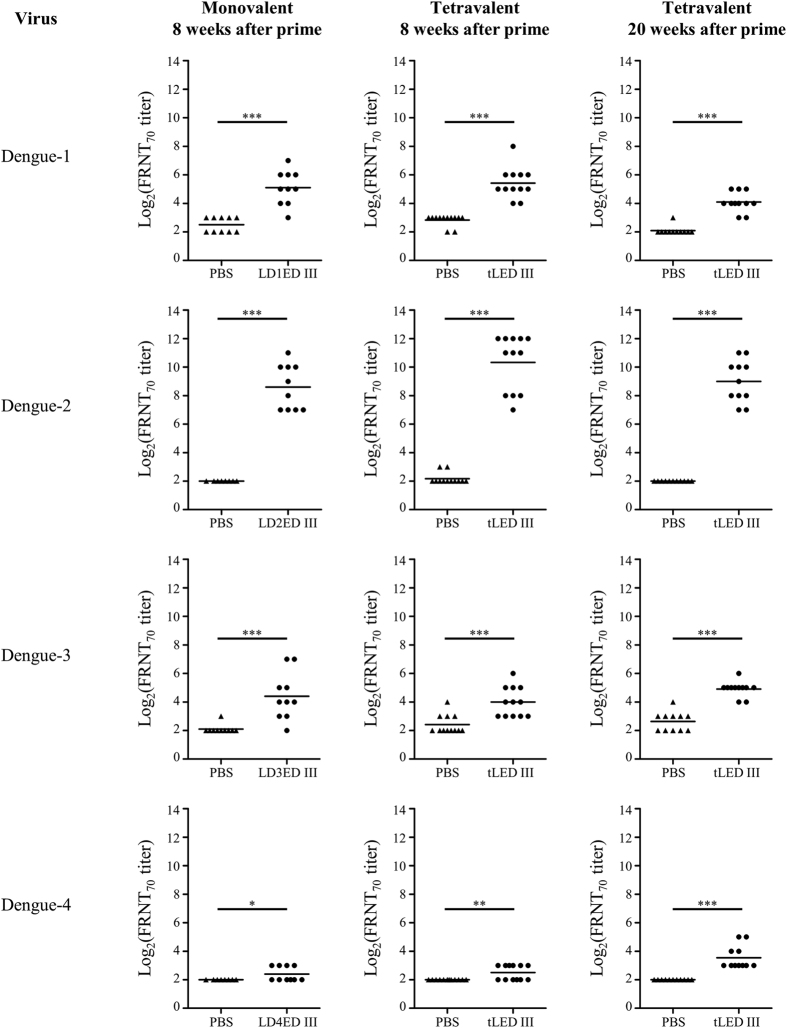
Neutralizing antibody responses against dengue virus in mice following immunization with monovalent or tetravalent recombinant lipidated dengue envelope protein domain III. Groups of BALB/c mice were immunized subcutaneously twice with a monovalent (10 μg recombinant lipidated dengue envelope protein domain III), a tetravalent (10 μg of each serotype of recombinant lipidated dengue envelope protein domain III) vaccine, or PBS at a 4-week interval. Serum samples were collected at the indicated time points. The neutralizing antibody capacity was determined by FRNT. The neutralizing antibody titer was calculated as the reciprocal of the highest dilution that resulted in a 70% reduction in FFU compared to control samples containing the virus alone. The neutralizing antibody titers were logarithmically transformed before statistical analyses. Statistical significance was determined by a two-tailed Mann Whitney test. *p < 0.05; **p < 0.01; ***p < 0.001. Symbols represent individual mice, and horizontal lines are mean values. The results shown are pooled from 2 experiments.

We next evaluated the capacity of the antibodies to neutralize dengue virus. Figure 3 shows the FRNT_70_ titers induced by monovalent and tetravalent vaccines. All animals immunized with the monovalent vaccines, except for the lipidated dengue-4 ED III (LD4ED III) vaccine candidate, elicited neutralizing antibodies against the homologous serotype at 8 weeks after the first immunization (FRNT_70_ titers >8). Mice inoculated with a tetravalent vaccine induced neutralizing antibodies against serotypes 1, 2, and 3 comparable to those induced by mice vaccinated with each homologous monovalent vaccine. The neutralizing antibodies against dengue-4 were low but detectable in mice receiving the tetravalent vaccine. Notably, the neutralizing antibodies were persistent and still detectable 20 weeks after the first vaccination. The geometric mean of neutralizing antibody titers (FRNT_70_) against serotypes 1, 2, 3, and 4 were 17, 512, 30, and 12, respectively.

**Figure 4:**
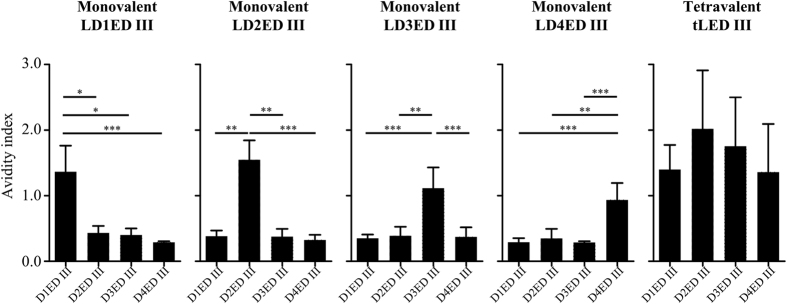
Analysis of IgG antibody avidity in mice immunized monovalent or tetravalent recombinant lipidated dengue envelope protein domain III. Groups of BALB/c mice were immunized subcutaneously twice with a monovalent (10 μg recombinant lipidated dengue envelope protein domain III) or a tetravalent (10 μg of each serotype of recombinant lipidated dengue envelope protein domain III) vaccine at a 4-week interval. Serum samples were collected at 8 weeks after the first immunization. Antibody avidity profiles were examined by ELISA. Data represent the mean ± standard deviation from total 10–12 mice of 2 experiments. Statistical significance was determined by Kruskal-Wallis test with Dunn’s multiple comparison test. *p < 0.05; **p < 0.01; ***p < 0.001.

Additionally, we examined the antibody avidity profiles of the serum samples at 8 weeks after the first immunization. As shown in Fig. 4, avidity index of antibody induced by individual monovalent lipidated ED III against heterotypic envelope protein domain III was less than 0.5 and significantly lower than that of the antibody against homotypic envelope protein domain III (avidity index >1.0). In contrast, tLED III induced high avidity index of antibody against all 4 serotypes of envelope protein domain III, which were comparable with each other. These results indicate the presence of a substantial concentration of high affinity antibodies against all 4 serotypes of envelope protein domain III in mice immunized with tLED III.

### The combination of the four serotypes of lipidated envelope protein domain III induces CD4^+^ and CD8^+^ T-cell responses in mice

**Figure 5:**
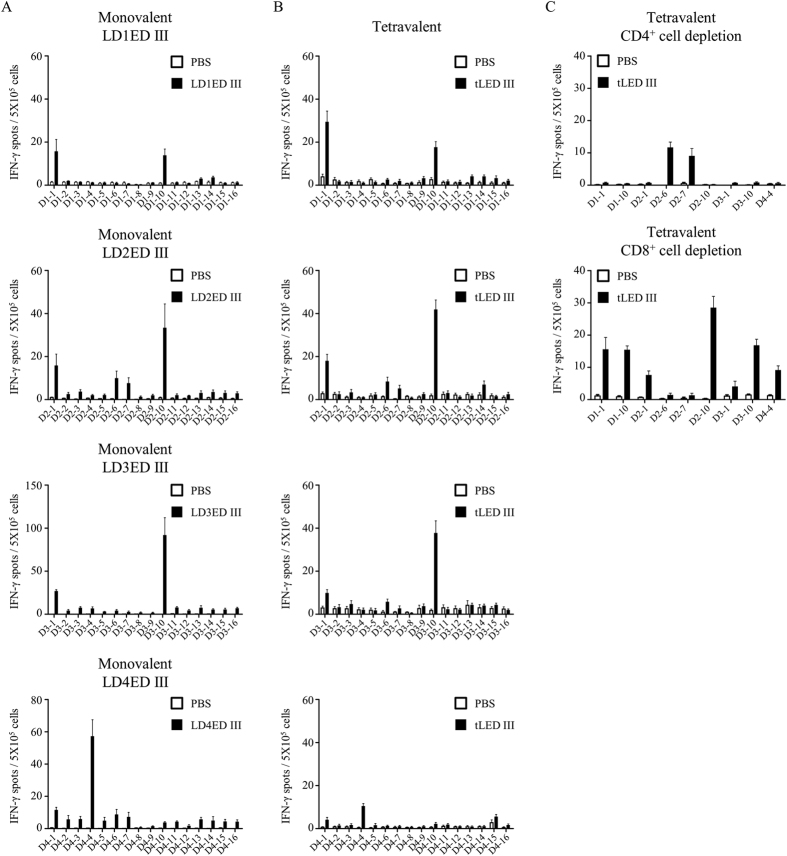
IFN-γ secretion in mice following immunization with monovalent or tetravalent recombinant lipidated dengue envelope protein domain III. Groups of BALB/c mice (n = 3–4) were immunized subcutaneously twice with a monovalent (10 μg recombinant lipidated dengue envelope protein domain III), a tetravalent (10 μg of each serotype of recombinant lipidated dengue envelope protein domain III) vaccine, or PBS at a 4-week interval. Splenocytes were prepared one week after the last immunization. A panel of 15-mer overlapping peptides that covered the entire sequence of each dengue envelope protein domain III was used to stimulate splenocytes. The frequencies of IFN-γ-producing cells in spleens were determined by mouse INF-γ ELISPOT kits. Data represent the mean ± standard error from total 6–8 mice of 2 experiments.

Groups of BALB/c mice were immunized with PBS, monovalent vaccines or tLDED III two times at a 4-week interval. Four weeks after the boost vaccination, splenocytes from vaccinated mice were assayed by ELISPOT to analyze the T-cell epitopes located on the envelope protein domain IIIs. A panel of 16 overlapping peptides for each serotype envelope protein domain III was used to stimulate INF-γ production. Splenocytes from BALB/c mice immunized with PBS induced a background level of INF-γ secretion for all the peptides stimulations (Fig. 5). A total of 9 dominant peptides were identified to induce INF-γ responses. Among them, 2 peptides (D1-1 and D1-10), 4 peptides (D2-1, D2-6, D2-7, and D1-10), 2 peptides (D3-1 and D3-10), and 1 peptide (D4-4) were identified from monovalent LD1ED III, LD2ED III, LD3ED III, and LD4ED III immunized mice, respectively (Fig. 5A). Splenocytes from tLDED III immunized mice also induced INF-γ production upon stimulation with these 9 dominant peptides (Fig. 5B). To further test whether the specific INF-γ production was CD4^+^ T-cell or CD8^+^ T-cell dependent in tLED III immunized mice, CD4-depleted and CD8-depleted splenocytes were used to assay INF-γ production after stimulation by the 9 dominant peptides individually. As shown in Fig. 5C, all specific IFN-γ responses disappeared in CD4-depleted cells after peptide stimulation except for stimulation by peptides D2-6 and D2-7. In contrast, all specific IFN-γ responses were still present in CD8-depleted cells after peptide stimulations except for stimulation by peptides D2-6 and D2-7. These results suggest that D2-6 and D2-7 peptides are recognized by CD8^+^ T cells, and the other 7 peptides are recognized by CD4^+^ T cells.

### The tetravalent formulation inhibits viremia levels in mice against the four serotypes of dengue virus

Mice are not the natural host of dengue virus. When infected with dengue virus, mice do not show dengue fever symptoms. While some immunodeficient mice are sensitive to the infection of dengue virus, they typically lack normal immune responses, making them unsuitable for vaccine evaluation. Reduction in the magnitude of viremia is thus considered to be a reliable indicator of *in vivo* dengue vaccine efficacy in mice. Challenging laboratory strains of immunocompetent mice with dengue-infected K562 cells led to transient viremia in these mice^42^. We adopted this simple method to evaluate the efficacy of virus clearance in vaccine immunized mice. BALB/c mice were immunized with tLED III twice at a 4-week interval. Eight weeks after the first immunization, the animals were individually challenged with K562 cells infected with each serotype of dengue virus. In parallel, PBS immunized mice were served as controls. Viral loads in the blood of tLED III-immunized mice were significantly lower than in that of PBS-immunized mice during the 4 to 32 hours after challenge. These results indicate that tLED III-immunized mice developed functional immune responses to clear all 4 serotypes of dengue virus from the circulation.

## Discussion

In our previous studies, we demonstrated that the performance of lipidated dengue envelope protein domain III is superior to its non-lipidated counterpart^38^,^39^,^40^,^41^. However, more than 55% of envelope protein domain III amino acid sequences are different across the 4 serotypes of dengue virus used to construct tLED III^27^. These sequence differences might influence the immune responses of the proteins derived from the individual serotypes. In the present study, we mixed equal amount of each dengue serotype of the lipidated envelope protein domain III as a tetravalent formulation and evaluated its immunogenicity in mice. In agreement with previous results using monovalent lipidated dengue envelope protein domain III^38^,^39^,^40^,^41^, mice immunized with tLED III could generate high and sustained antibody responses in the absence of exogenous adjuvant formulation (Fig. 1). Importantly, mice immunized with tLED III could also elicit neutralizing antibodies against all 4 serotypes of dengue virus. These neutralizing antibodies were sustained for up to 20 weeks after the first vaccination (Fig. 3). Induction of long-lasting antibody responses is a hallmark of a good vaccine. These results suggest that tLED III is a potential dengue vaccine formulation.

It has been shown that dengue envelope protein domain III-based subunit vaccines formulated with CpG plus aluminum hydroxide^28^ or Freund’s adjuvant^25^ elicit IgG1, IgG2a, and IgG2b, but not IgG3, antibody responses. However, sera from mice infected with live dengue virus exhibited a much more diverse IgG subclass response, including IgG1, IgG2a, IgG2b, and IgG3. Sera obtained from tLED III immunized mice also contained IgG1, IgG2a, IgG2b, and IgG3 antibodies. These results suggest that tLED III without exogenous adjuvant formulation is able to induce a response of a diverse subclass of IgGs, which is similar to the response to dengue virus. This scenario is in accord with our previous observations with a monovalent vaccine candidate, LD3ED III^39^.

Dengue disease is a complex viral disease that is caused by 4 serotypes of dengue virus. Viral interference was reported in the approach with the live-attenuated virus where a mix of four monovalent dengue vaccine candidates was used^43^,^44^. Titers of neutralizing antibodies were dominated by a particular serotype. This dominance was associated with the replication potential of the vaccine candidate, but the detailed mechanism is unclear. The occurrence of interference in tetravalent live-attenuated virus formulation may cause failure in providing full protection for all 4 serotypes^45^. Subunit vaccines are not like live-attenuated virus vaccines because subunit vaccines do not replicate *in vivo*; this suggests that tLED III may not cause interference by different replication efficiencies of the vaccine candidates. In this study, tLED III induced different neutralizing antibody titers than those of the 4 serotypes but comparable to those induced by individual monovalent formulation (Fig. 3). These results suggest that the difference in titers of the neutralizing antibodies among the 4 serotypes of dengue virus induced by tLED III is not the cause of interference.

It has been shown that antibody avidity correlates with a neutralizing capacity in natural infections of human by dengue virus^46^. Moreover, an increased severity of dengue diseases is associated with a lower antibody avidity at later time-points after infection^47^. Therefore, antibody avidity should be an important index to evaluate vaccine candidates. The individual monovalent formulation only induced high affinity antibodies against homotypic envelope protein domain III but not heterotypic envelope protein domain III. Meanwhile, antibodies raised by individual monovalent formulation can not neutralizing heterotypic virus (Supplementary Table S1). However, tLED III elicited high and comparable affinity antibodies against all 4 serotypes of envelope protein domain III (Fig. 4). These antibodies also can neutralizing 4 serotypes of dengue virus. To further examine whether lipidated ED III raised antibodies can recognize native dengue virus, ELISA was performed by using dengue virus coated 96-well plates (Supplementary Figure S1). Similar to avidity profiles, antibodies induced by tLED III recognized all 4 serotypes of dengue virus. In contrast, antibodies induced by the individual monovalent formulation recognized homotypic virus, only slightly cross reacted to heterotypic virus at high concentration of sera (1:33). These results suggest that lipidated ED III in monovalent formulation is prone to serotype-specific antibody responses and tetravalent formulation recognizes all 4 serotypes of dengue virus. Altogether, these results not only indicate that high affinity antibodies against the 4 serotypes of envelope protein domain III induced by tLED III may bring advantage to the host but also support that there is no significant interference of tLED III upon the induction of antibody responses.

T-cell responses are very important for humoral immune responses. Both CD4^+^ and CD8^+^ T cells have been shown to play a protective role against dengue viral infection^48^,^49^,^50^. However, T-cell responses elicited by tLED III have not been fully characterized. To further dissect the T-cell epitopes within the dengue envelope protein domain III in tLED III-immunized mice, synthetic overlapping peptides covering each serotype envelope protein domain III were used to stimulate IFN-γ secretion. All 9 dominant peptides identified from monovalent lipidated envelope protein domain III were able to stimulate IFN-γ secretion in tLED III-immunized mice (Fig. 5A,B). These results suggest that tLED III-immunized mice generate a wide spectrum in the T-cell epitope profiles. This diversity covered the epitopes generated by each monovalent formulation-immunized mice. The 9 dominant peptides can be localized to 4 regions. One epitope region on envelope protein residues 325 to 345 (D2-6 and D2-7) is recognized by CD8^+^ T cells (Fig. 5C) and includes an L^d^-restricted CD8^+^ T-cell epitope (SPCKIPFEI)^51^. Another region is on envelope protein residues 313 to 327 (D4-4) and is recognized by CD4^+^ T cells (Fig. 5C), which has been reported in our previous study^52^. The other two are on envelope protein residues 295 to 309 (D1-1, D2-1, and D3-1) and 349-363 (D1-10, D2-10, and D3-10) and are both recognized by CD4^+^ T cells (Fig. 5C), consistent with previous reports^52^,^53^,^54^. CD4^+^ T cells reacted to peptides derived from 4 serotypes of dengue virus in vaccinated mice. In contrast, CD8^+^ T cells only reacted to peptides derived from dengue-2 virus in vaccinated mice. In this study, 15-mer peptides with 9 overlapping amino acids but not 8-10-mer peptides were used for stimulation in ELISPOT assays. We can not rule out that some CD8^+^ T-cell epitopes are embedded within the longer 15-mer peptides. Although nonstructural proteins 3, 4B, and 5 are the dominant regions recognized by CD8^+^ T cells in human infection with dengue virus^55^ or vaccination with live attenuated tetravalent dengue vaccine^56^, some CD8^+^ T-cell epitopes are within ED III^55^,^56^. ED III is the only region used as a vaccine candidate in this study. It is possible that tLED III may induce CD8^+^ T-cell responses and further boost after infection in human. Our results show that tLED III in the absence of exogenous adjuvant formulation is efficient to elicit both CD4^+^ and CD8^+^ T-cell responses against all 4 serotypes of envelope protein domain III.

**Figure 6:**
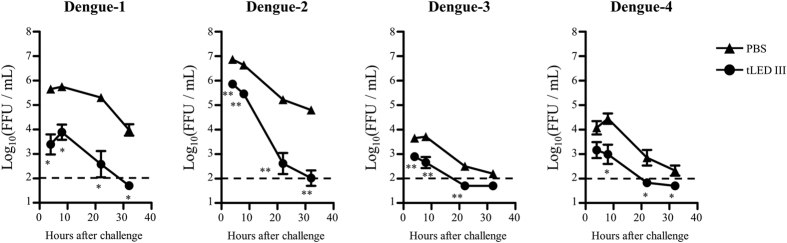
Reduction of viremia levels in vaccinated mice after they were challenged with dengue virus. Groups of BALB/c mice (n = 4–6) were immunized subcutaneously twice with a tetravalent (10 μg of each serotype of recombinant lipidated dengue envelope protein domain III) vaccine or PBS at a 4-week interval. Mice were intraperitoneally challenged with dengue-infected K562 cells at 8 weeks after the first vaccination. The mice were bled at 4, 8, 22, and 32 hours after the challenge. The infective viral titers in plasma were determined by focus-forming assays using BHK-21 cells. The viral titers below the detection limit (2.0 log_10_ FFU/mL), as indicated as a dash line, were assigned a value of 1.7. The infective viral titers were logarithmically transformed before statistical analyses. Data represent the mean ± standard error. Statistical significance was determined by a two-tailed Mann Whitney test. *p < 0.05; **p < 0.01.

Viremia levels were only detectable within 10 min after injection of dengue virus by the intravenous route in mice^57^. In this study, animals were challenged with dengue virus-infected K562 cells. This approach can prolong viremia up to 1 day^42^. In addition, using a dengue virus-infected myeloid lineage cell line (K562) is analogous to the type of cells thought to be infected humans with dengue virus^58^. In this model, dengue virus was produced by infected-K562 cells in the peritoneal cavity. Several factors may affect the viremia levels after challenge, including but not limited to the frequencies of infected-K562 cells at challenge, the efficiency of production of dengue virus by infected-K562 cells, the replication potential of dengue virus, and capability of dengue virus in the peritoneal cavity entering the circulation. These results reflect different viremia levels among 4 serotypes of dengue virus in PBS-immunized mice. We demonstrate that all 4 serotypes of dengue virus were cleared from the circulation after challenge in tLED III-immunized mice, which were faster than the clearance observed in the PBS groups (Fig. 6). These results suggest that tLED III induces functional immune responses in eliminating the 4 serotypes of dengue virus *in vivo*.

In summary, tLED III can induce high antibody titers that are stable over a prolonged period of time without exogenous adjuvant formulation. Meanwhile, these antibodies are of high affinity and can neutralize all 4 serotypes of dengue virus. Importantly, tLED III elicit multivalent T-cell responses with a wide spectrum of T-cell epitope profiles against all 4 serotypes of dengue virus. These represent important features on dengue vaccine development. Our results reveal that tLED III can induce a broad spectrum of immunity in mice, which lays a foundation for further detailed clinical studies.

## Materials and Methods

### Virus and recombinant proteins

The laboratory-adapted virus Dengue-1/Hawaii, dengue-2/PL046, dengue-3/H-087, dengue-4/H241 were used for this study. All the viruses were kindly provided by Yi-Ling Lin of the Institute of Biomedical Sciences, Academia Sinica, Taiwan. Virus propagation was implemented in C6/36 cell. Viral titers were determined by focus-forming assays with BHK-21 cells as previously described^27^. The preparation of 4 serotypes of lipidated ED III was previously described^38^,^39^,^40^,^41^. All recombinant proteins were lyophilized and stored at −20 °C until use.

### Experimental mice and immunization

BALB/c mice (female) were acquired from the National Laboratory Animal Breeding and Research Center (Taipei, Taiwan). The animals were kept at the Laboratory Animal Center of the National Health Research Institutes (NHRI). All of the animal experiments were approved and conducted according to the guidelines of the Animal Committee of the NHRI. All mice were immunized with the monovalent vaccine (10 μg/0.2 mL/dose) or tetravalent vaccine (5 μg of each monovalent vaccine/0.2 mL/dose) via subcutaneous injection at 6–8 weeks of age. Mice that were injected with PBS alone (without vaccine candidates) served as controls. All animals were immunized 2 times at a 4-week interval with the same regimen. Blood samples were obtained by tail bleeding from each mouse at different time points as indicated. All sera were prepared and stored at −20 °C until use.

### Measurement of antibody titers and avidity

The presence of antigen-specific IgG in the sera were determined by titrating the samples. A 3-fold serial dilution (starting at 1:33) of each sample was prepared. The recombinant D1ED III, D2ED III, D3ED III, and D4ED III were coated onto 96-well plates. Bound IgG, IgG1, IgG2a, IgG2b, or IgG3 were detected with HRP-conjugated goat anti-mouse IgG Fc (ICN Cappel), biotin-conjugated rabbit anti-mouse IgG1 (Thermo Fisher Scientific Inc., USA), IgG2a (Thermo Fisher Scientific Inc., USA), IgG2b (Thermo Fisher Scientific Inc., USA), or IgG3 (Fitzgerald Industries International Inc.), respectively. After washing, streptavidin-HRP conjugate (Pharmingen) was added for the detection of IgG1, IgG2a, IgG2b, or IgG3. A substrate, 3,3′,5,5′-tetramethylbenzidine (TMB), was added for color development. The absorbance was measured with an ELISA reader at 450 nm. The ELISA end-point titer was defined as the serum dilution that produced an OD value of 0.5. All titers were obtained from the titration curve by interpolation, unless the OD value was less than 0.5 at the starting dilution (1:33). Determination of antibody avidity was based on antigen-specific IgG dissociation induced by the ammonium thiocyanate (a chaotropic agent), as previously described^39^.

### Focus reduction neutralization tests (FRNT)

Serum samples were heat-inactivated before testing. A 2-fold serial dilution (starting at 1:8) of each sample was prepared. The neutralizing capacity of each sample was determined as previously described^27^. The neutralizing antibody titer FRNT_70_ was determined as the reciprocal of the highest dilution that resulted in a 70% decrease in focus-forming units (FFUs) compared with control samples containing the virus alone. The neutralizing antibody titer was designated as 4 when the titer was less than the starting dilution (1:8) for calculation purpose.

### ELISPOT assays

Mice were sacrificed, and splenocytes were prepared one week after the last immunization. The frequency of IFN-γ-producing cells was determined by mouse IFN-γ ELISPOT kits (PB Pharmingen). All assays were conducted according to the manufacturer’s procedures. In brief, capture antibodies were coated onto 96-well plates with PVDF membranes (Millipore) and incubated at 4 °C for 18 hours. The plates were washed twice and blocked with RPMI medium supplemented with feta bovine serum (10%) for 1 hour to prevent nonspecific binding in later steps. The splenocytes were seeded at a concentration of 5 × 10^5 ^cells/well with a panel of 16 15-mer peptides with 9 overlapping amino acids derived from each serotype of ED III (2 μg/mL)^52^. Triplicate wells were set up for each stimulation. PBS, control peptides, or concanavalin A (5 μg/mL) were included as controls in parallel. After stimulation for 2 days at 37 °C in a 5% CO_2_ humidified incubator, the cells were removed from the plates by washing three times with 0.05% (w/v) Tween 20 in PBS. A 100-μL aliquot of biotinylated detection antibody was added to each well. The plates were incubated at 37 °C for 2 hours. The washing steps were repeated. After a 45-minute incubation at room temperature with the avidin-horseradish peroxidase complex reagent, the plates were washed three times with 0.05% (w/v) Tween 20 in PBS and then three times with PBS alone. A 100-μL aliquot of 3-amine-9-ethylcarbazole (Sigma-Aldrich) staining solution was added to each well to develop the spots. The reaction was stopped after 1 hour by placing the plates under tap water. The spots were counted using an ELISPOT reader (Cellular Technology Ltd.).

### Preparation of dengue virus-infected K562 cells

K562 cells (1 × 10^7^/0.5 mL) mixed with 0.5 mL serum-free RPMI medium (containing 0.2 × 10^7^ FFU dengue-1, 1 × 10^7^ FFU dengue-2, 0.5 × 10^7^ FFU dengue-3, or 3.3 × 10^7^ FFU dengue-4). After incubation at 37 °C for 2 hours, K562 cells were collected by brief centrifugation and resuspended in 20 mL of 5% FBS RPMI medium. Cells were cultured at 37 °C for 3–4 days. The infected-K562 cells (1 × 10^7^/10 mL) were collected and mixed with unifected-K562 cells (1 × 10^7^/30 mL) for a second round of infection. After 2 days, the cells were harvested and used for the injection into mice. Meanwhile, cultured fluids were collected and monitored for viral titers. Viral titers for dengue-1, dengue-2, dengue-3, and dengue-4 were 3.0 × 10^6^, 2.8 × 10^7^, 3.0 × 10^4^, and 4.2 × 10^3 ^ffu/mL, respectively.

### Animal challenge

The immunized mice were intraperitoneally injected with 5 × 10^7^ dengue virus-infected K562 cells that were suspended in 0.5 mL of serum-free RPMI medium at 8 weeks after the first immunization^39^,^41^. Blood samples were obtained at 4, 8, 22, and 32 hours after challenge. The blood (0.2 mL) was immediately mixed with 0.02 mL of 3.8% sodium citrate pre-chilled on ice. The plasma was isolated, and the viremia level was evaluated using focus-forming assays with BHK-21 cells. Any infective titers below the limit of detection (2.0 log_10_ FFU/mL) were assigned a value of 1.7 for calculation purpose.

### Statistical analyses

The means, standard deviations, standard errors, and statistics analyses were calculated using GraphPad Prism software version 5.02 (GraphPad Software, Inc.). The analysis of the antibody avidity data was performed with Kruskal-Wallis test with Dunn’s multiple comparison tests. The neutralizing antibody data and viremia levels from the challenge assay in mice were analyzed by two-tailed Mann Whitney tests. Differences with *p* < 0.05 were considered to be statistically significant.

## Additional Information

**How to cite this article**: Chiang, C.-Y. *et al*. Immunogenicity of a novel tetravalent vaccine formulation with four recombinant lipidated dengue envelope protein domain IIIs in mice. *Sci. Rep*. **6**, 30648; doi: 10.1038/srep30648 (2016).

## Supplementary Material

Supplementary Information
