# Supplementary material for: Immunogenicity of a novel tetravalent vaccine formulation with four recombinant lipidated dengue envelope protein domain IIIs in mice
Source: Sci Rep. 2016 Jul 29;6:30648. doi: 10.1038/srep30648 (PMC4965760; doi:10.1038/srep30648)
Supplement: Supplementary Information [file srep30648-s1.pdf]

Supplementary Information for:

Immunogenicity of a novel tetravalent vaccine formulation with four recombinant lipidated dengue envelope protein domain IIIs in mice

Chen-Yi Chiang[1,†], Chien-Hsiung Pan[1, 2,†], Mei-Yu Chen[1], Chun-Hsiang Hsieh[1],Jy-Ping Tsai[1], Hsueh-Hung Liu[1], Shih-Jen Liu[1, 2], Pele Chong[1, 2], Chih-Hsiang Leng[1, 2, *] and Hsin-Wei Chen[1, 2, *]

[1]National Institute of Infectious Diseases and Vaccinology, National Health Research Institutes, 35 Keyan Road, Zhunan, Miaoli 350, Taiwan, Republic of China
[2]Graduate Institute of Immunology, China Medical University, Taichung, Taiwan, Republic of China

[*]**Corresponding authors:** Hsin-Wei Chen, E-mail: chenhw@nhri.org.tw, Tel: 886-3-724-6166 ext 37706, Fax: 886-3-758-3009 or Chih-Hsiang Leng, E-mail: leoleng@nhri.org.tw, Tel: 886-3-724-6166 ext 37711, Fax: 886-3-758-3009
[†]these authors contributed equally to this work.

Contents:

Supplementary Table S1
Supplementary Figure S1

Supplementary Table S1. Neutralizing antibody titers ($FRNT_{70}$) in mice immunized with monovalent formulations.

	Vaccine			
	LD1ED III	LD2ED III	LD3ED III	LD4ED III
Dengue-1	$2^5, 2^3, 2^4, 2^4, 2^5$	$<2^3, <2^3, <2^3, <2^3, <2^3$	$<2^3, <2^3, <2^3, <2^3, <2^3$	$<2^3, <2^3, <2^3, <2^3, <2^3$
Dengue-2	$<2^3, <2^3, <2^3, <2^3, <2^3$	$2^{10}, 2^9, 2^7, 2^7, 2^7$	$<2^3, <2^3, <2^3, <2^3, <2^3$	$<2^3, <2^3, <2^3, <2^3, <2^3$
Dengue-3	$<2^3, <2^3, <2^3, <2^3, <2^3$	$<2^3, <2^3, <2^3, <2^3, <2^3$	$2^4, <2^3, 2^3, 2^3, 2^5$	$<2^3, <2^3, <2^3, <2^3, <2^3$
Dengue-4	$<2^3, <2^3, <2^3, <2^3, <2^3$	$<2^3, <2^3, <2^3, <2^3, <2^3$	$<2^3, <2^3, <2^3, <2^3, <2^3$	$2^3, 2^3, <2^3, <2^3, 2^3$

Supplementary Figure S1.

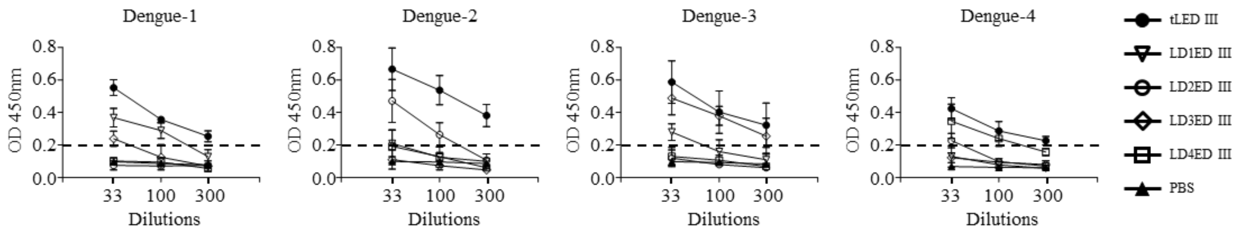

Supplementary Figure S1. Induction of antibody responses against 4 serotypes of dengue virus in mice immunized with a monovalent or tetravalent recombinant lipidated dengue envelope protein domain IIIs. Groups of BALB/c mice (n=5-6) were immunized subcutaneously twice with a monovalent vaccine (10 µg recombinant lipidated dengue envelope protein domain III), a tetravalent vaccine (10 µg of each serotype of recombinant lipidated dengue envelope protein domain III) or PBS at a 4-week interval. Serum samples were collected at 8 weeks after the first immunization. IgG antibodies against dengue-1, dengue-2, dengue-3 and dengue-4 virus were evaluated by ELISA, respectively. Data represent mean ± standard deviation. Dashed lines indicate 2-fold of mean background levels (OD450nm= 0.2).
